# From Pathology to Precision Medicine in Anaplastic Large Cell Lymphoma Expressing Anaplastic Lymphoma Kinase (ALK+ ALCL)

**DOI:** 10.3390/cancers9100138

**Published:** 2017-10-16

**Authors:** Michael T. Werner, Qian Zhang, Mariusz A. Wasik

**Affiliations:** Department of Pathology and Laboratory Medicine, University of Pennsylvania, Philadelphia, PA 19104, USA; miwerner@mail.med.upenn.edu (M.T.W.); qian2@mail.med.upenn.edu (Q.Z.)

**Keywords:** anaplastic lymphoma kinase, anaplastic large cell lymphoma, NPM-ALK, ALK+ ALCL, precision medicine

## Abstract

Anaplastic large cell lymphoma expressing anaplastic lymphoma kinase (ALK+ ALCL) is a distinct subtype of non-Hodgkin lymphoma. In this review, we discuss the historical findings that led to its classification as a unique disease, despite its varied clinical presentation and histology. We discuss the molecular mechanisms underlying ALK+ ALCL pathology and the questions that remain in the field. Finally, we visit how decades of ALK+ ALCL research has yielded more precise drugs that hold promise for the future.

## 1. Anaplastic Large Cell Lymphoma in Historical Context

Anaplastic Large Cell Lymphoma (ALCL) represents 10–15% of non-Hodgkin lymphoma in children but can manifest throughout adulthood [1]. Though it is a rare disease, its impact should not be overlooked. Relapses and chemotherapy-related toxicities present a tremendous burden to patients, their families and the healthcare system.

Like many other malignancies, ALCL was initially defined not by its genotype, but by its phenotype. Its identification began with the cloning of the diagnostic antibody Ki-1, which recognizes a surface protein on a subset of Hodgkin and non-Hodgkin lymphomas [2]. The Ki-1 antibody was consistently reactive against poorly classified non-Hodgkin lymphomas that collectively exhibited abundant cytoplasm, large irregular nuclei and a tendency toward intrasinusoidal invasion. These tumors had diverse morphologies, including common type, lymphohistiocytic, small-cell and Hodgkin-like variants, but were unified in their reactivity with Ki-1 and often expressed T cell antigens. Collectively, they were termed ALCL [3]. The target of Ki-1 was later identified as CD30, a cytokine receptor from the tumor necrosis factor receptor family [4]. Though CD30 can be expressed on Reed-Sternberg cells characteristic of Hodgkin lymphoma, in the context of non-Hodgkin lymphoma, its expression became pathognomonic for ALCL. Based on growing clinical and cytological evidence for a distinct disease, ALCL was included in the Kiel lymphoma classification [5].

A molecular understanding of ALCL began with the observation that a large subset of ALCL cases harbored a t(2;5) (p23;q35) chromosomal translocation [6]. Subsequent cloning of the translocation identified two genes: nucleophosmin 1 (NPM1) and a new kinase that was named anaplastic lymphoma kinase (ALK) [7]. ALK is a receptor tyrosine kinase whose expression is normally restricted to neural progenitor cells during development [8,9]. The t(2;5) translocation yields an abundantly expressed chimeric protein containing the oligomerization motif of NPM1 and the kinase domain of ALK [7,8,10]. The NPM-ALK homodimer cross-phosphorylates itself leading to its persistent kinase activation [11,12]. The NPM-ALK fusion is by far the most common translocation product in ALCL [13], though several other oncogenic ALK fusion partners with similar mechanisms have been identified [14]. Based on this additional genetic evidence, the World Health Organization (WHO) recognized ALK+ ALCL in 2008 [15].

Per the revised WHO lymphoma classification in 2016, four distinct entities of ALCL currently are recognized: (1) ALCL, ALK+, (2) ALCL, ALK-negative, (3) primary cutaneous ALCL and (4) breast implant-associated ALCL [16]. These classifications are based on a combination of clinical, histopathological and genetic attributes. For example, ALK+ and ALK-negative ALCL are systemic diseases with multi-nodal involvement usually of intra-abdominal and mediastinal lymph nodes, whereas primary cutaneous ALCL and breast implant-associated ALCL are more localized and less aggressive [1]. The latter two diseases rarely, if ever, express ALK. However, typical features of most ALK+ and ALK-negative ALCL tumors include the presence of “hallmark cells,” which are large cells with kidney-shaped nuclei and a peri-nuclear eosinophilic region, and the essentially universal expression of the CD30 antigen.

Though there is strong evidence that NPM-ALK is critical in the lymphomagenesis of ALK+ ALCL, one cannot ignore the above observation that a large subset of ALCL, particularly in older adults, lacks ALK expression. One study suggests that aberrant expression of oncogenes near the t(2;5) breakpoint, prior to the translocation, promotes cell growth [17]. This could potentially explain some of the morphological and transcriptional consistencies between ALK+ and ALK-negative ALCL. However, more recent studies show that ALK-negative ALCL tumors often contain translocations involving IRF4/DUSP22 [18,19], TP63 [20] and less frequently ROS and TYK2, which are related to ALK and JAK kinases, respectively [21]. In addition, gain of function mutations in JAK1 and STAT3 genes have been reported in ALK-negative cases [21]. In sum, these alternative mechanisms better explain the pathogenesis of ALK-negative ALCL.

## 2. ALK Biology in the Crosshairs of Medicine

Chemotherapy remains the predominant treatment for patients with ALCL. As a rare disease, conducting clinical trials that carefully assess different treatment strategies for ALK+ ALCL is relatively difficult. Furthermore, overall survival rates are quite high for the standard chemotherapy regimens, in particular for ALK+ ALCL in children and young adults; however, late relapses are relatively common even in this group. The drawbacks of chemotherapy—its toxicities leading to sterility and secondary malignancies among other impairments—are also well recognized. These side effects are particularly important in the pediatric population [1]. Therefore, the sands are shifting in favor of more targeted approaches based on the two hallmark features of ALK+ ALCL: CD30 expression and ALK activity. CD30 is normally expressed on activated immune cells, but it is also universally expressed in ALCL with ALK shown to promote activation of the CD30 gene [22]. Brentuximab vedotin is a monoclonal antibody against CD30 that delivers a microtubule inhibitor specifically to CD30+ cells. It received FDA approval as a frontline single-agent therapy in ALCL [23]. Similarly, ALK itself provides an ideal target for therapy. It is not expressed in normal tissues outside of development [13], and thus is a cancer-specific kinase. Moreover, ALK drives essentially all oncogenic and metastatic pathways in ALK+ ALCL [24], the foremost being STAT3 [25,26]. Multiple generations of ALK inhibitors are at various stages of clinical development. These inhibitors are studied mostly in the context of more prevalent ALK+ cancers for which clinical trials are easier to populate, particularly ALK+ lung carcinoma [27]. However, ALCL-inclusive trials and case series of ALCL patients treated with the first-generation ALK inhibitor crizotinib have yielded remarkably positive results, particularly in the pediatric population [28,29,30]. In a recent study by the Children’s Oncology Group (COG), 21 out of 26 pediatric patients exhibited a complete response to ALK inhibition using crizotinib as a front-line monotherapy [31]. We anticipate that a current COG-sponsored trial testing crizotinib in combination with chemotherapy will demonstrate even higher efficacy (NCT01606878). Another arm of the ongoing COG-sponsored clinical trial combines chemotherapy with brentuximab vendotin. Similar to the chemotherapy/crizotinib combination arm, the chemotherapy/brentuximab combination is anticipated to yield high efficacy exceeding that of the agents administered alone.

Despite the preliminary successes reported for ALK kinase inhibition in ALK+ ALCL, it is worth noting that resistance mutations similar to those seen in other ALK+ cancers have been reported both in patients [30] and in vitro [32,33]. Not surprisingly, these ALK kinase mutations alter the sensitivities of ALCL cells to various ALK inhibitors [34]. Amin et al. [33] demonstrated that several of their ALK-resistant sub-clones had robust up regulation of NPM-ALK gene copy number reflected in elevated mRNA and protein expression levels. These inhibitor-resistant sub-clones were paradoxically addicted to ALK inhibitor, as removal of inhibitor resulted in cell death. The authors attribute this phenomenon to unbalanced ALK activity that is toxic upon inhibitor removal. Therefore, a cocktail of ALK inhibitors, as compared to a single inhibitor, may prove to be most effective if used upfront to preempt selection for resistant clones that would lead to relapse.

**Figure 1:**
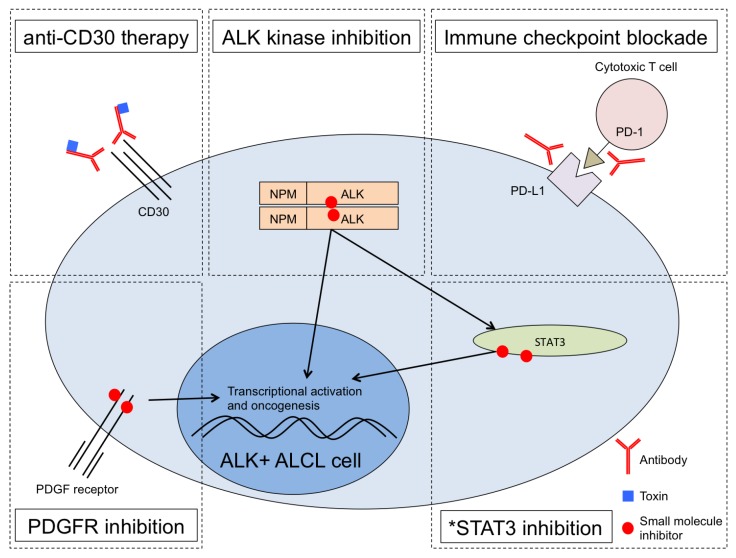
Multiple therapeutic pathways in ALK+ ALCL can be combined for optimized treatment. Anti-CD30 treatment with brentuximab vedotin is FDA-approved [23]. ALK inhibitors such as the first-generation drug crizotinib are in various stages of clinical development with strong preliminary clinical results in ALCL [28,29,30]. PD-1 checkpoint blockade resulted in complete remission for a patient with chemotherapy-resistant and ALK inhibitor-resistant ALCL relapse [35]. Experimental treatment using the PDGFR inhibitor imatinib resulted in a full remission of a patient in relapse [36]. *STAT3 inhibition is an area of active research in ALCL and other cancers but remains experimental, limited so far in ALCL to pre-clinical studies.

Furthermore, multipronged treatment strategies involving ALK inhibition, anti-CD30 therapy and other agents should yield more durable clinical responses (Figure 1). Remarkably, a patient with ALK+ ALCL that developed resistance to both chemotherapy and ALK inhibition experienced complete remission upon T-cell checkpoint blockade therapy targeting PD-1 [35]. Similar results were seen in an ALK+ ALCL patient treated with imatinib [36], which though developed as a BCR-ABL kinase inhibitor also inhibits enzymatic activity of PDGFRA, PDGFRB, and c-KIT kinases Importantly, PDGFRB was shown to be a direct target of JUN/JUNB complex downstream of NPM-ALK, and imatinib efficacy was shown to have a positive correlation with PDGFRB expression in mice bearing ALK+ ALCL tumors [36]. These single case observations coupled with the recently proven efficacy of ALK inhibition monotherapy strongly suggest that rationally designed, potentially individualized combination therapies with ALK inhibition at their backbone should prove highly effective. Existing and new experimental models of ALK+ ALCL [37] should demonstrate the efficacy of such combinations.

In the meantime, many groups are using high-throughput approaches to identify novel drug targets in ALK+ ALCL [24]. We present two recent examples. First, a phosphoproteomic screen identified the Wiskott-Aldrich syndrome protein (WASp) as a direct NPM-ALK substrate [38]. WASp is a central regulator of actin polymerization, with well-characterized roles in normal T cells. Phosphorylation of WASp by NPM-ALK enhances ALCL tumor growth and invasion, and WASp is required for tumor growth. Given that an inhibitor to the closely related protein N-WASP has been reported [39], a new line of investigation into WASp inhibition in models of ALCL could prove beneficial. Second, a CRISPR-based loss of function screen revealed the cytokine receptor IL-31Rβ as an additional drug target in ALK+ ALCL [40]. It is of note that IL-31R has a previously demonstrated role in the pathogenesis of cutaneous T cell lymphoma [41,42] and follicular B cell lymphoma [43]. This suggests that targeting the IL-31/IL-31R axis, either with antibodies that block the receptor-ligand interaction or with small molecule inhibitors of the downstream signaling pathways (predominately STAT3), could be additionally beneficial. 

## 3. Targeting STAT3 in ALK+ ALCL

One of the emerging stories stemming from the molecular characterization of NPM-ALK function is a consistent role for STAT3 signaling in the pathogenesis of ALCL. STAT3 is phosphorylated in normal cells by members of the JAK family in response to cytokine-cytokine receptor interaction. Phospho-STAT3 translocates into the nucleus, and functions as a transcriptional activator. Several lines of evidence support its role in ALCL. First, STAT3 is required for ALK-mediated tumorigenesis in lymphoma [26]. Second, a striking correlation exists between ALK-dependent and IL-2-dependent transcriptional changes in ALK+ ALCL [44]. Third, convergent mutations of JAK1 and/or STAT3 itself in ALK-negative ALCL also result in STAT3 activation [21]. STAT3 is thus an attractive drug target (Figure 1). However, targeting STAT3 has proven difficult [45] and so other potential targets in this pathway have garnered attention. 

In dissecting the role of the JAK/STAT pathway in ALK+ ALCL, a controversy has emerged as to whether NPM-ALK can bypass JAK to phosphorylate STAT3 directly. If JAK3, or other members of the family, were an intermediary of NPM-ALK signaling, then it would function as a suitable drug target. Initial evidence suggested that this was indeed the case. JAK3 was found to be constitutively active in ALCL cells and to physically interact with NPM-ALK [46,47,48]. Accordingly, JAK inhibition revealed a dose-dependent loss of STAT3 activity [46]. However, these reports were in contrast to a previous study in which an NPM-ALK mutant unable to bind JAK3 could still phosphorylate STAT3 [49]. A potential explanation for this discrepancy is that the JAK inhibitors used in the Amin et al. [46] study had off-target binding to the kinase domain of ALK thus ultimately reducing STAT3 phosphorylation independently of JAK3. This explanation was further supported by later work which showed that the JAK3 inhibitors used in the Amin et al. [46] study could directly inhibit NPM-ALK kinase activity in vitro and that JAK3 was, in fact, unnecessary for STAT3 phosphorylation [50].

Recent technological advances should help resolve this controversy and further elucidate targetable pathways in ALK+ ALCL. First, with many ALK inhibitors now available as research tools, a thorough analysis of JAK kinase function upon ALK inhibition could be conducted. If NPM-ALK phosphorylates STAT3 via JAK, then treatment with structurally different ALK inhibitors should always simultaneously reduce phosphorylation of JAKs and STAT3. Second, using CRISPR-based genome editing tools, JAK knockout ALCL cells could be generated to determine the contribution of JAKs to NPM-ALK function and ALCL cell growth. Finally, to identify additional drug targets potentially outside of the NPM-ALK-JAK/STAT pathway, a more general CRISPR-screen could be completed in ALK+ ALCL cells.

## 4. Conclusions

In summary, ALCL initially appeared as an enigmatic disease. However, as the clinical, histological, and genetic evidence mounted, a distinct entity of T cell lymphoma for the ALK-expressing subtype emerged. The pathogenesis of ALK-negative ALCL strongly parallels that of the ALK+ subtype in that intracellular cytokine signaling pathways are often engaged. Based on these findings, new and more precise therapies have been developed. It appears likely that such precision medicine will become the mainstay of treatment in ALCL.
